# Review Article: Fibrosis Reversal in Metabolic Dysfunction‐Associated Steatohepatitis—The Promise of Emerging Therapeutics

**DOI:** 10.1111/apt.70921

**Published:** 2026-08-17

**Authors:** Malek Ayoub, Tsubasa Tsutsumi, Mary E. Rinella

**Affiliations:** ^1^ Division of Gastroenterology and Hepatology University of Chicago Pritzker School of Medicine Chicago Illinois USA

## Abstract

**Background:**

Metabolic dysfunction‐associated steatohepatitis (MASH) is a leading indication for liver transplantation, and fibrosis stage most strongly predicts clinical outcomes. Pharmacologic treatment is now available with resmetirom and semaglutide holding conditional approval.

**Aims:**

To describe the therapeutic landscape for MASH with a focus on anti‐fibrotic impact.

**Methods:**

Narrative review of Phase 2 and 3 trials, meta‐analyses, epidemiological data, and trial registries.

**Results:**

Historically, drugs targeting downstream fibrogenic or inflammatory pathways have had limited success in advanced development, whereas those improving metabolic dysfunction have had fared better. Fibrosis improvement by at least one stage was seen with resmetirom in up to 26% versus 14% placebo after 52 weeks and with semaglutide in 37% versus 22% after 72 weeks. Several agents in development (e.g., fibroblast growth factor 21 analogues, as well as dual and triple incretin agonists, pan‐peroxisome proliferator‐activated receptor agonist) report encouraging Phase 2 results, with Phase 3 trials ongoing. Compensated cirrhosis requires a distinct approach related to its fibrosis heterogeneity and burden of portal hypertension. This requires a delicate balance between efficacy and event rates that is further complicated by current regulatory constraints.

**Conclusions:**

Fibrosis improvement in MASH is attainable by available drugs with further promise from emerging therapies. The link between treatment induced histological improvement or improvement in noninvasive tests and reduction in clinical outcomes remains unproven. A rich therapeutic pipeline with the promise of combination regimens and increased focus non‐invasive endpoints suggests that the future looks bright for patients living with fibrotic MASH.

## Graphical abstract

Metabolic dysfunction‐associated steatohepatitis is the second commonest US indication for liver transplantation but remains largely undiagnosed and untreated. Fibrosis stage is the strongest predictor of major adverse liver outcomes, with decompensation rates and transplant‐free survival diverging sharply between F3 and F4, while cardiovascular disease and extrahepatic malignancy remain the leading causes of death. Two conditionally approved drugs, resmetirom and semaglutide, now demonstrate that hepatic fibrosis is pharmacologically reversible, and a broad pipeline of incretin‐based, FGF21 and pan‐PPAR agents is advancing. Translating histological benefit into reduced decompensation, transplantation and death is the outstanding question for the field.

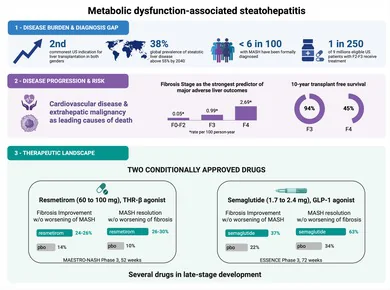

## 1 Introduction

Medicine has a strong track record of changing the natural history of liver disease when it can identify and neutralise the underlying disease driver. The most striking example remains hepatitis C. The arrival of direct‐acting antiviral (DAA) therapies transformed what had previously been a slowly progressive and largely untreatable condition into one that is curable in the great majority of patients. Listings for hepatitis C, which had been rising steadily through the early 2000s, peaked around 2012/2013 and have fallen steadily every year since, tracking closely with the availability of highly effective DAA regimens from 2014 onward [1].

Metabolic dysfunction‐associated steatohepatitis (MASH) has not yet cleared that bar. Metabolic dysfunction‐associated steatotic liver disease (MASLD) now affects an estimated 38% of the global population, with projections suggesting prevalence will exceed 55% by 2040 [2]. MASH is now the second most common indication for liver transplantation in both men and women in the United States [3], a position it has reached while HCV listings have continued to fall. The contrast is a direct reflection of what effective, widely deployed therapy can do to the natural history of a liver disease. Chronic liver disease and cirrhosis account for 52,274 deaths per year in the United States, ranking 11th among all causes of mortality, and MASH is responsible for a growing and likely underestimated share of that burden.

The last two years have brought major therapeutic progress. The conditional approvals of resmetirom in March 2024 [4] and semaglutide in August 2025 [5] confirm that pharmacological reversal of hepatic fibrosis in MASH is achievable, and that both liver‐directed and upstream metabolic strategies can produce meaningful histological benefit within the timeframes of current trials. Fibrosis improvement was observed in approximately one in three to four treated patients at 52 to 72 weeks, and whether these effects further reduce fibrosis or improve outcomes with longer treatment duration remains an open and important question. MAESTRO‐NASH [6], the 54‐month outcomes study of resmetirom, will be the first trial designed to address it, with results anticipated in early 2028. Yet by the end of 2025, resmetirom had been prescribed to more than 36,250 patients, roughly 0.4% of the estimated nine million Americans with F2 or F3 MASH who would be potentially eligible for treatment [7]. Approval and population‐level impact are not the same, and the gap between them in MASH remains substantial. This review examines the biological, pharmacological, and operational determinants of that gap, and considers what therapeutic strategies and non‐invasive monitoring tools will be needed to close it.

## 2 Fibrosis Stage, Clinical Risk, and the Rationale for Pharmacological Intervention

Fibrosis stage has emerged as the single strongest independent predictor of liver‐related morbidity and mortality in MASH. A meta‐analysis of over 17,000 patients with biopsy‐confirmed disease confirmed a stepwise increase in the risk of liver‐related events and all‐cause mortality with each unit increase in fibrosis stage [8]. The clinical differences between stages are not linear. The incidence of new‐onset hepatic decompensation is 0.05 per 100 person‐years at F0 to F2, rising to 0.99 at F3 and 2.69 at F4 [9]. Ten‐year transplant‐free survival is approximately 94% at F3 and falls to 45% at F4 [8].

These data highlights those that should be targeted for pharmacological intervention, though treatment approach may differ across the fibrosis spectrum, depending on the mechanism of action of the drug. Metabolic phenotype alone does not fully capture long‐term risk: steatotic liver disease‐associated genetic variants, including risk alleles in PNPLA3 and TM6SF2 and the protective HSD17B13 variant, further modify susceptibility to hepatic and extrahepatic outcomes [10].

For patients with F0 or F1 disease, liver‐related event risk is low, and the therapeutic priority is metabolic optimisation, as at earlier disease stage, effectively managing the root cause of the disease (metabolic dysfunction) is likely to avert the development of further liver injury and fibrosis. Sustained weight loss, improved glycemic control, and cardiovascular risk reduction are the foundations of management at this stage, supported by lifestyle modification and, where indicated, incretin‐based therapy [11]. As fibrosis advances into Stage 2, and more so at F3, patients are most likely to benefit from an intervention that has direct antifibrotic potential, ideally in addition to favourable metabolic benefits. The risk of further progression to cirrhosis carries with it an increased risk of hepatic decompensation and death. Once cirrhosis (F4) is established, the risk of liver related outcomes increases substantially. Approximately 1.15 million Americans currently have MASH cirrhosis, a figure projected to approach 2.2 million by 2050 [12]. Annual decompensation rates in compensated cirrhosis are 5 to 7 per 100 person‐years [13]. These rates illustrate the potential population‐level importance of identifying and treating patients with advanced fibrosis. However, the magnitude by which pharmacological fibrosis improvement will reduce decompensation, transplantation, hepatocellular carcinoma, or death remains uncertain. Longitudinal and post hoc trial data support an association between fibrosis regression, lower liver stiffness, and reduced liver‐related events, but completed MASH trials have not yet established a direct quantitative relationship between drug‐induced fibrosis improvement and clinical‐event reduction.

Awareness and identification of patients with clinically relevant disease remains an important barrier. Fewer than 6% of patients with MASH have been formally diagnosed, and only a fraction of those is receiving treatment [2]. Non‐invasive tools have established prognostic utility and are increasingly used to direct pharmacological evaluation [14]. However, at current treatment rates, the population‐level impact of even highly effective pharmacotherapy will remain negligible relative to the burden of undiagnosed and untreated disease. A further contributor to this diagnostic gap is lean MASLD, which is increasingly recognised and frequently missed by screening strategies that rely on metabolic risk factors [15]. It is also important to recognise that the performance of widely used non‐invasive tests is not uniform across the at‐risk population. FIB‐4 in particular has reduced rule‐out reliability in populations with a high prevalence of advanced fibrosis, such as patients with Type 2 diabetes, because the absolute number of false‐negative results increases as pretest risk rises. Its interpretation is also confounded by age, with reduced specificity in older adults and a higher cut‐off recommended in those older than 65 years [16, 17]. A sequential approach using FIB‐4 followed by VCTE or ELF, with MRE reversed for indeterminate cases or specialist assessment, is therefore preferred when pretest risk remains high or FIB‐4 results are uncertain [18].

## 3 Targeting Fibrogenesis in Isolation: Rationale, Evidence, and Limitations

The biological case for directly interrupting the fibroinflammatory cascade in MASH is clear. Hepatic stellate cell activation, extracellular matrix deposition, and the inflammatory and apoptotic signals that trigger and sustain progressive fibrosis are mechanistically well characterised, and multiple nodes within these pathways represent legitimate pharmacological targets supported by robust preclinical data [19, 20, 21, 22, 23]. The field committed substantial resources to this approach over the past two decades. Despite engaging targets that are mechanistically well‐validated in MASH pathogenesis, these programs ultimately did not meet their prespecified histologic endpoints in pivotal trials, an outcome that is informative as much for what it reveals about the underlying disease biology as for what it indicates about the individual drugs themselves (Figure 1).

**FIGURE 1:**
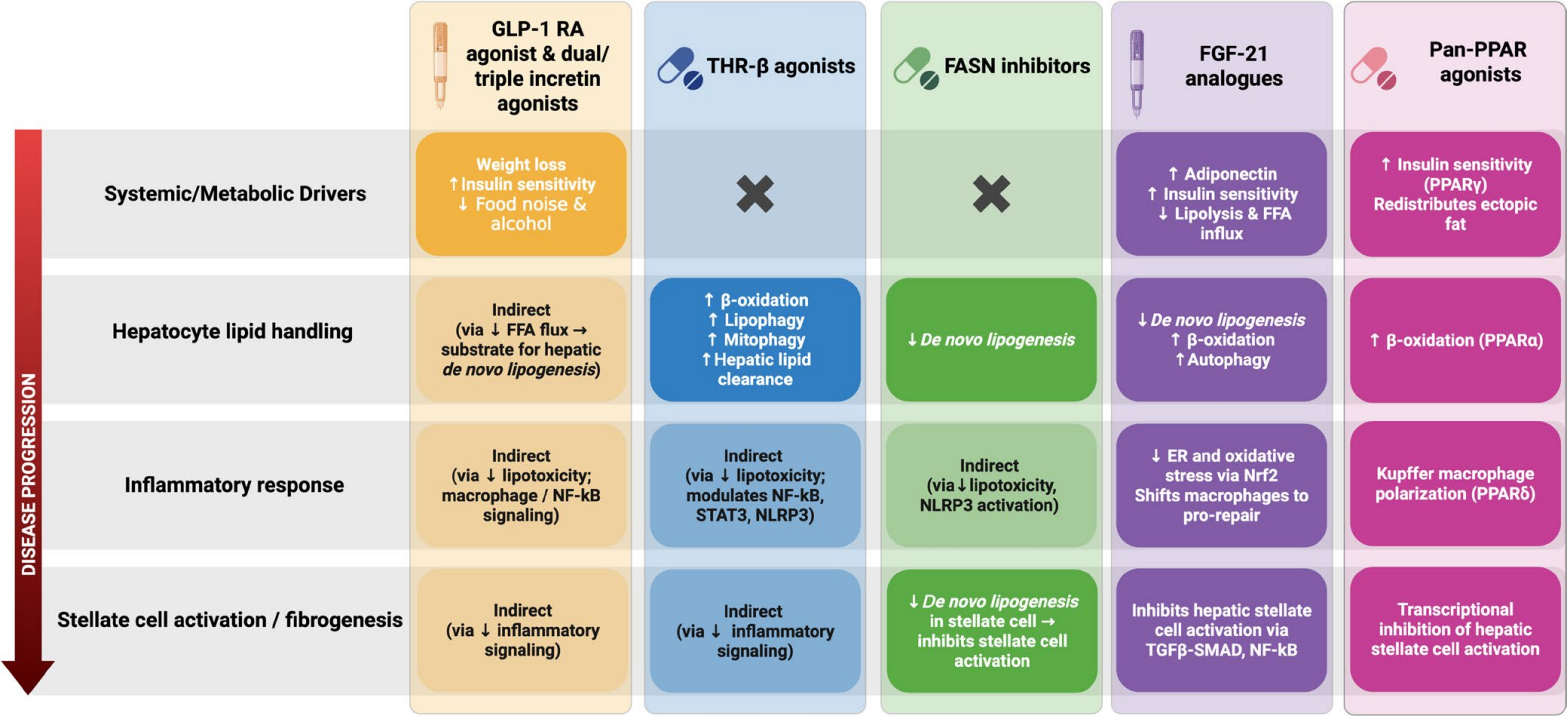
Direct and indirect effects of conditionally approved and/or late‐stage development drug classes along the MASH disease progression axis. Rows show successive steps in MASH pathogenesis, from upstream systemic metabolic drivers down to stellate cell activation and fibrogenesis. Columns show the main drug classes with conditional approval in non‐cirrhotic MASH and/or in late‐stage development.

Simtuzumab, a monoclonal antibody targeting lysyl oxidase homologue 2, produced no significant histological benefit in Phase 3 trials in patients with bridging fibrosis or compensated cirrhosis due to MASH [21]. Selonsertib, an apoptosis signal‐regulating kinase 1 inhibitor designed to interrupt hepatocyte injury and downstream stellate cell activation, failed to meet its primary endpoints in both the STELLAR‐3 and STELLAR‐4 trials [20]. Cenicriviroc, a dual CCR2 and CCR5 antagonist targeting inflammatory monocyte recruitment, failed in the AURORA Phase 3 trial [19]. The pattern across these failures was strikingly consistent, though the primary reasons were not uniform. For example, in the case of simtuzumab, preclinical support was insufficient; for selonsertib, the earlier phase trial was not convincingly positive and progression to Phase 3 was premature [24]; for cenicriviroc, a secondary fibrosis signal in Phase 2b did not replicate in the Phase 3 trial, which was terminated at a pre‐specified interim analysis due to lack of efficacy on the primary histological endpoint [25].

The most plausible underlying explanation for failure in advanced clinical trials is not fully due to the pharmacology of these individual agents but in the conceptual framework underlying their development. MASH is not primarily a disease of fibrogenesis. It is a disease of systemic metabolic dysfunction in which fibrosis represents the end result of sustained lipotoxic injury (Figure 1). Furthermore, targeting the fibrogenic response in isolation without dampening the root cause is unlikely to render optimal results [11]. Redundant fibrogenic pathways, compensatory biological mechanisms, and the pharmacokinetic challenge of achieving adequate tissue penetration in an increasingly fibrotic liver all compound this fundamental limitation. Trial‐level factors compound these biological limitations: high placebo response rate across MASH trials, and interobserver variability in central histological reading of fibrosis stage adds outcomes noise that reduces statistical power [26].

## 4 Mechanistic Determinants of Histological Efficacy: Evidence From Approved and Emerging Therapies

The agents that have achieved the most robust histological benefit share a defining mechanistic characteristic: each addresses the metabolic substrate of hepatic injury rather than targeting the fibrogenic or inflammatory response it generates in isolation. Whether that metabolic correction operates at the level of intrahepatic lipid disposal, systemic insulin resistance, or adipose‐liver crosstalk varies by drug class, but the convergence on metabolic pathophysiology as the essential therapeutic target is consistent across approved agents, and the emerging pipeline (Figure 1).

### 4.1 Resmetirom: Restoration of Intrahepatic Lipid Homeostasis

Resmetirom, the first drug conditionally approved for the treatment of MASH with F2 or F3 fibrosis [4], is a liver‐selective thyroid hormone receptor beta (THR β) agonist whose dominant mechanism is the restoration of mitochondrial fatty acid oxidative capacity within hepatocytes. In MASH, intrahepatic hypothyroidism characterised by impaired T4‐to‐T3 conversion results in reduced THRβ signalling, decreased β‐oxidation, and progressive lipid accumulation. By selectively activating THRβ, resmetirom induces a hepatocyte transcriptional response that markedly increases mitochondrial fatty acid oxidation and promotes lipophagy and mitophagy, thereby directly reducing the intrahepatic lipotoxic burden that drives downstream inflammation and fibrogenesis [27]. Critically, these effects are hepatocyte‐intrinsic: resmetirom does not produce meaningful weight loss or systemic insulin sensitisation, which distinguishes it mechanistically from both incretin‐based therapies and which makes it particularly relevant for patients with significant fibrosis independent of their metabolic phenotype.

In the MAESTRO‐NASH Phase 3 trial, resmetirom at 80 mg and 100 mg led to fibrosis improvement of at least one stage without worsening of MASH in 24.2% and 25.9% of patients respectively, versus 14.2% in the placebo group (*p* < 0.001 for both doses) [6]. MASH resolution was achieved in 25.9% and 29.9% respectively, versus 9.7% receiving placebo (*p* < 0.001 for both doses) [6]. A secondary analysis of MAESTRO‐NASH further indicated that the histological and biomarker effects of resmetirom were generally consistent across patients receiving GLP‐1 receptor agonists and other background diabetes therapies, a finding of direct relevance to the combination strategies discussed below [28]. Cardiovascular disease is the leading cause of death in patients with MASH. Importantly, resmetirom achieved a dose dependent reduction in LDL cholesterol of 13.6% to 16.3% relative to placebo [6], Whether this LDL‐c reduction translates into a measurable cardiovascular event benefit in the MASH population remains unproven. The magnitude of LDL‐c lowering is substantially smaller than that typically achieved with statin therapy, and concurrent statin dose adjustments were permitted in MAESTRO‐MASH. Dedicated cardiovascular outcome data are not yet available. The ongoing MAESTRO‐NASH‐OUTCOMES trial (NCT05500222 [29]) is evaluating its potential in compensated cirrhosis, with results expected in 2027 (Table 1).

**TABLE 1:** Key Phase 3 clinical trials in MASH targeting fibrosis improvement and liver‐related outcomes.

Trial/NCT	Population	Primary endpoint(s)	Timepoint	Est. primary completion (CT.gov)
MAESTRO program—resmetirom (selective THR‐β agonist)—Conditionally approved for non‐cirrhotic MASH				
MAESTRO‐NASH NCT03900429	Biopsy‐confirmed non‐cirrhotic MASH	Histology: MASH resolution without worsening of fibrosis; ≥ 1‐stage fibrosis improvement without worsening of MASH Outcomes: all‐cause mortality, liver transplant, and significant hepatic events (including hepatic decompensation events [ascites, encephalopathy, or gastroesophageal variceal haemorrhage], histological progression to cirrhosis, and a confirmed increase of MELD score from < 12 to ≥ 15)	52 weeks 54 months	Completed Q1 2028
MAESTRO‐NASH‐OUTCOMES NCT05500222	MASH compensated cirrhosis	Outcomes: all‐cause mortality, liver transplant, ascites, hepatic encephalopathy, gastroesophageal variceal haemorrhage, and confirmed increase of MELD score from < 12 to ≥ 15 due to liver disease	36 months	Q1 2027
MAESTRO‐NAFLD NCT04197479	MASLD	Safety and tolerability	52 weeks	Completed
MAESTRO‐NAFLD‐OLE NCT04951219	MASLD	Safety and tolerability	52 weeks	Completed
ESSENCE program—semaglutide (GLP‐1 receptor agonist)—Conditionally approved for non‐cirrhotic MASH				
ESSENCE NCT04822181	Biopsy‐confirmed non‐cirrhotic MASH	Interim histology endoints: MASH resolution without worsening of fibrosis; ≥ 1‐stage fibrosis improvement without worsening of MASH Outcomes for final approval: cirrhosis free survival	72 weeks 240 weeks	Completed Q2 2029
NATiV program—lanifibranor (pan‐PPAR agonist)				
NATiV3 NCT04849728	Biopsy‐confirmed non‐cirrhotic MASH	Histology: MASH resolution without worsening of fibrosis; ≥ 1‐stage fibrosis improvement without worsening of MASH	72 weeks	Q4 2026
ENLIGHTEN program—pegozafermin (FGF21 analogue)				
ENLIGHTEN‐Fibrosis NCT06318169	Biopsy‐confirmed non‐cirrhotic MASH	Histology: MASH resolution without worsening of fibrosis; ≥ 1‐stage fibrosis improvement without worsening of MASH Outcomes	52 weeks 5 years	Q1 2029 ?
ENLIGHTEN‐Cirrhosis NCT06419374	MASH compensated cirrhosis	Histology: Fibrosis regression is defined as improvement in fibrosis by ≥ 1 stage Outcomes: Time to First Occurrence of Disease Progression as Measured by Composite of Protocol‐Specified Clinical Events	24 months 5 years	Q3 2028 Q3 2031
SYNCHRONY program—efruxifermin (Fc‐FGF21 fusion)				
SYNCHRONY Histology NCT06215716	Biopsy‐confirmed non‐cirrhotic MASH	Histology: MASH resolution without worsening of fibrosis; ≥ 1‐stage fibrosis improvement without worsening of MASH Outcomes: Based on time from randomisation to the first clinical event including evidence of disease progression, liver decompensation events, liver transplantation or eligibility for liver transplantation, and all‐cause mortality	52 weeks 240 weeks	Q4 2032? ?
SYNCHRONY Real‐World NCT06161571	MASLD	Safety and tolerability	52 weeks	Q3 2026
SYNCHRONY Outcomes NCT06528314	MASH compensated cirrhosis	Histology: Cohort 1 only: ≥ 1 stage improvement in fibrosis and no worsening of steatohepatitis Outcomes: Time from randomisation to first occurrence of disease progression as measured by composite of protocol‐specified clinical events	96 weeks 5 years	Q4 2029 ?
ZENITH program—efimosfermin alfa (long‐acting Fc‐FGF21)				
ZENITH‐1 NCT07221227	Biopsy‐confirmed non‐cirrhotic MASH	Histology: MASH resolution without worsening of fibrosis; ≥ 1‐stage fibrosis improvement without worsening of MASH Outcomes: Liver‐related outcome will be comprised of all‐cause mortality; transplantation; occurrence of significant hepatic events	52 weeks 48 months	Q4 2031 ?
ZENITH‐2 NCT07221188	MASLD	Safety and tolerability	52 weeks	Q3 2028
LIVERAGE program—survodutide (GLP‐1/glucagon dual agonist)				
LIVERAGE NCT06632444	Biopsy‐confirmed non‐cirrhotic MASH	Histology: MASH resolution without worsening of fibrosis; ≥ 1‐stage fibrosis improvement without worsening of MASH Outcomes: Progression to cirrhosis is defined as histological fibrosis score CRN F4. MELD = Model for End‐stage Liver Disease CSPH = clinically significant portal hypertension	52 weeks 7 years	Q4 2031 ?
LIVERAGE‐Cirrhosis NCT06632457	MASH cirrhosis	Outcomes: all‐cause mortality, liver transplant, hepatic decompensation events, worsening of MELD score to ≥ 15 and progression to CSPH	4.5 years	Q3 2029
SYNERGY program—tirzepatide/retatrutide (incretin‐based, parallel arms)				
SYNERGY‐OUTCOMES NCT07165028	MASLD/MASH enriched with NITs for advanced disease	Composite: progression to cirrhosis, development of large oesophageal varices, gastric varices or development of varices needing treatment, development of ascites, development of hepatic encephalopathy, evidence of active or recent variceal haemorrhage, increase in model for end‐stage liver disease (MELD) from ≤ 12 to ≥ 15, liver transplantation, all‐cause mortality	224 weeks	Q3 2030

### 4.2 Semaglutide: Upstream Metabolic Disease Control

Semaglutide, the 2nd conditionally approved drug that achieved accelerated approval for F2/F3 MASH [5], achieved a similar fibrosis benefit after 72 weeks, through a very distinct mechanism. As a GLP1 receptor agonist administered as a once‐weekly subcutaneous injection, it reduces hepatic lipid influx principally through enhanced satiety, weight loss, suppression of adipose tissue lipolysis, and improvement in insulin sensitivity. GLP1 receptors are not expressed on hepatocytes. However, recent experimental data indicate that GLP‐1 receptors are present on liver sinusoidal endothelial cells (LSECs) and may mediate weight‐loss‐independent hepatoprotection [30]. The hepatic benefits of semaglutide are therefore predominantly mediated through systemic metabolic improvement. Mediation analyses from both the Phase 2 and Phase 3 ESSENCE trials suggest that some of the anti‐fibrotic histologic benefit may be independent of the weight loss it produces, possibly involving extrahepatic anti‐inflammatory signalling in hepatic macrophages [31].

In the ESSENCE Phase 3 interim analysis of 800 participants with F2 or F3 MASH, semaglutide 2.4 mg once weekly achieved MASH resolution without worsening of fibrosis in 62.9% of treated patients versus 34.3% receiving placebo, and fibrosis improvement by at least one stage in 37.0% versus 22.4% (*p* < 0.001, for both) [32, 33] (Table 1). In patients with more advanced fibrosis or incomplete metabolic response to incretin‐based therapy, a liver‐directed agent addressing intrahepatic lipotoxicity directly may be required as an adjunct or alternative.

### 4.3 The Expanding Incretin Landscape: Dual and Triple Agonists in Development

Building on the Phase 3 validation of semaglutide, a wave of next‐generation incretin‐based therapies with enhanced metabolic potency is advancing through late‐phase development. The addition of glucose‐dependent insulinotropic polypeptide receptor agonism (GIP) to GLP‐1 receptor agonism, as in tirzepatide, produced MASH resolution in 44% to 62% versus 10% in the placebo group, and fibrosis improvement in 51% to 55% versus 30% in the placebo group in a Phase 2 trial, with mean weight losses of 10.7% to 15.6% versus 0.8% in the placebo group over 52 weeks [34]. In a head‐to‐head randomised trial in adults with obesity and no Type 2 diabetes, tirzepatide produced greater relative weight loss than semaglutide (20.2% vs. 13.7% at 72 weeks), providing the first direct evidence that GIP receptor co‐agonism confers additional metabolic potency beyond GLP1‐agonism alone [35]. Whether more pronounced weight loss will translate into superior hepatic outcomes in the MASH population, which is enriched with Type 2 diabetes and in whom weight loss responses tend to be more attenuated, remains to be addressed by the ongoing Phase 3 program (NCT07165028 [36]) (Table 1).

The addition of glucagon receptor agonism (GCGR) to GLP‐1 activity introduces a mechanistically distinct and potentially important dimension [37]. Since glucagon receptors are expressed abundantly in hepatocytes, the addition of glucagon agonism drives hepatic fatty acid oxidation and energy expenditure through pathways that are partially independent of systemic weight loss [38]. Beyond β‐oxidation, glucagon receptor signalling in hepatocytes inhibits de novo lipogenesis via PKA‐mediated inactivation of acetyl‐CoA carboxylase, reduces hepatic triglyceride and VLDL secretion, and engages amino acid catabolism as part of the liver–α‐cell axis, mechanisms that collectively reduce the pool of lipotoxic intermediates driving hepatocellular injury [39]. Because glucagon simultaneously opposes GLP‐1's insulinotropic effect, the relative potency at each receptor is a critical design parameter: insufficient glucagon activity forfeits the hepatic delipidating benefit, whereas excessive glucagon activity increases the risk of hyperglycemia [40]. The published GLP‐1 to glucagon activity ratios of clinical candidates vary widely, approximately 1:1 for pemvidutide, 5:1 for cotadutide, and 8:1 for survodutide, and are thought to shape both efficacy and glycemic tolerability across the class. Survodutide, a dual GLP‐1 and glucagon receptor agonist, produced MASH improvement in 47% to 62% versus 14% in the placebo group, and fibrosis improvement in 34% to 36% versus 22% in the placebo group across dose groups in a 48‐week Phase 2 trial [41], and has entered a Phase 3 outcomes program (Table 1). Pemvidutide, designed as a balanced 1:1 GLP‐1 and glucagon receptor agonist, led to MASH resolution in 52% to 58% versus 20% in the placebo group at 24 weeks (*p* < 0.0001), meeting its primary endpoint, though the co‐primary endpoint of fibrosis improvement by conventional histology at 24 weeks was not met in the intention‐to‐treat analysis (33% and 36% versus 28% placebo) [42]. At 48 weeks, pemvidutide achieved statistically significant reductions in the enhanced liver fibrosis score and liver stiffness measurement relative to placebo, providing non‐invasive evidence of continued antifibrotic activity with extended treatment, and Phase 3 development is planned for 2026 (Table 1).

Retatrutide is a triple agonist of the GIP, GLP‐1, and glucagon receptors, but importantly, its in vitro pharmacology is GIPR‐dominant rather than glucagon‐weighted. Published EC50 values, the half‐maximal effective concentration, that is, the drug concentration at which 50% of the maximal receptor response is achieved, with lower values indicating higher potency, are approximately 0.06 nM at GIPR, 0.78 nM at GLP‐1R, and 5.8 nM at GCGR, yielding roughly balanced GLP‐1 and glucagon activities with markedly greater potency at GIPR [43]. This profile distinguishes retatrutide mechanistically from more glucagon‐weighted dual agonists such as pemvidutide and survodutide, and likely contributes to its large weight‐loss effect with preserved glycemic tolerability. In a 48‐week Phase 2a sub‐study in patients with MASLD and Type 2 diabetes, retatrutide achieved reductions in liver fat content of up to 82% relative to placebo [44]. The triple‐agonist mechanism is pharmacologically the most comprehensive of any agent in this class, and the weight loss data from the obesity program without Type 2 diabetes, which showed mean reductions of 22% to 24% at 48 weeks, position retatrutide as potentially the most potent metabolic drug among all incretin‐based therapies in development. However, weight loss is likely to be attenuated in the MASH population where Type 2 diabetes is highly prevalent.

### 4.4 FGF21 Analogues: Bridging Systemic and Intrahepatic Mechanisms

Fibroblast growth factor 21 analogues act on the liver through two complementary mechanisms: an indirect, adiponectin‐mediated mechanism and a direct hepatic mechanism. The indirect mechanism dominates systemically: FGF21 signalling through FGFR1c and β‐Klotho on adipose tissue sensitises adipocytes to insulin, restrains lipolytic release of free fatty acids, and drives secretion of adiponectin, which in turn appears responsible for a substantial share of the downstream benefits on hepatic metabolism, inflammation, and fibrogenesis [45]. The direct mechanism operates within the liver itself: in hepatocytes, FGF21 activates Nrf2‐dependent antioxidant defences and autophagy, relieving endoplasmic reticulum stress and limiting apoptosis. The bulk of the antifibrotic effects of FGF21 analogues is therefore likely to be indirect, operating through adipose‐derived adiponectin and reduced hepatocellular lipotoxicity. A direct effect on hepatic stellate cells has been suggested in vitro, but in vivo evidence is limited, and the relative contribution of any direct stellate‐cell action remains uncertain [45].

Efruxifermin, the most clinically advanced FGF21 analogue, demonstrated statistically significant fibrosis improvement versus placebo at 96 weeks in the HARMONY Phase 2b trial in patients with F2 to F3 MASH. At 96 weeks, fibrosis improvement without worsening of MASH was observed in 30% (28 mg) and 49% (50 mg) versus 19% in the placebo group in the modified intention‐to‐treat population (*p* = 0.003 for the 50 mg dose). MASH resolution without worsening of fibrosis was achieved in 40% (28 mg, *p* = 0.02) and 37% (50 mg, *p* = 0.039) respectively versus 19% in the placebo group. The progressive separation of treatment and placebo curves from 24 to 96 weeks is noteworthy, suggesting that FGF21 agonism may require sustained treatment duration to fully engage its antifibrotic mechanisms [46]. Efruxifermin has entered the Phase 3 SYNCHRONY program in pre‐cirrhotic and cirrhotic MASH (Table 1).

Pegozafermin, a long acting glycopegylated FGF21 analogue, was evaluated in the Phase 2b ENLIVEN trial in patients with F2 to F3 MASH. At 24 weeks, fibrosis improvement of at least one stage without worsening of MASH was achieved in 26% (30 mg QW) and 27% (44 mg Q2W) of treated patients versus 7% receiving placebo, and MASH resolution without worsening of fibrosis in 23% (30 mg QW) and 26% (44 mg Q2W) versus 2% in the placebo group [47]. Pegozafermin has advanced into the Phase 3 ENLIGHTEN program, including ENLIGHTEN‐Fibrosis in F2/F3 MASH and ENLIGHTEN‐Cirrhosis in compensated cirrhosis (NCT6318169 [48]; NCT06419374 [49]).

Efimosfermin‐alfa, a long‐acting FGF21 analogue administered once monthly, has also shown encouraging preliminary efficacy. In a 24‐week Phase 2 trial in patients with F2 to F3 MASH, fibrosis improvement of at least one stage without worsening of MASH occurred in 45% (300 mg monthly) of treated patients versus 21% receiving placebo, accompanied by high rates of MASH resolution (65% in the treated patients versus 29% in the placebo [50]). Its once‐monthly subcutaneous administration is a potentially advantageous feature for chronic therapy, and Phase 3 development is planned.

As a class, FGF21 analogues raise several safety considerations relevant to long‐term use. The most common adverse events are gastrointestinal symptoms, particularly nausea and diarrhoea together with injection‐site reactions, which are generally mild to moderate. Immunogenicity is an additional consideration, as anti‐drug antibodies have been reported and their potential to attenuate drug exposure or efficacy during prolonged therapy is not yet fully resolved. Changes in bone mineral density and bone‐turnover markers have been reported inconsistently across analogues and warrant continued monitoring and more detailed assessment. Long‐term comparative safety data for the class overall remain limited, an important caveat as these agents move toward chronic‐disease use [45].

### 4.5 Other Agents Consistent With the Metabolic Anchoring Principle

Denifanstat, a fatty acid synthase inhibitor, targets the committed step in hepatic de novo lipogenesis by blocking palmitate synthesis from malonyl‐CoA, directly reducing the intrahepatic generation of saturated fatty acids that sustain lipotoxic injury. In the FASCINATE‐2 Phase 2b trial, denifanstat led to MASH resolution and fibrosis improvement compared to placebo (*p* < 0.02). A notable side effect that is likely an on‐target effect on hair follicles is alopecia, which occurred in 19% in treated patients in the Phase 2b [51]. Planned Phase 3 data would be important to more accurately characterise the impact of Denifanstat on hair loss and how this may be mitigated.

Lanifibranor, a pan‐peroxisome proliferator‐activated receptor agonist, engages PPARα, PPARγ, and PPARδ simultaneously, addressing hepatic lipid oxidation, adipose insulin sensitisation, and anti‐inflammatory macrophage polarisation through a single compound. In the NATIVE Phase 2 trial, lanifibranor met its primary endpoint of SAF improvement over placebo (*p* < 0.001) (Table 1), with Phase 3 data expected by Q4 2026.

## 5 The Challenge of Cirrhosis: A Distinct Therapeutic and Regulatory Frontier

Treatment for patients living with cirrhosis represents the highest unmet need, as these patients face the most immediate risk of major adverse liver outcomes (MALO). The annual risk of hepatic decompensation at this stage is approximately 5% to 7%, and once decompensation occurs, five‐year survival falls below 50% [13]. Cirrhosis, however, is not a single disease state but a broad spectrum along which both prognosis and therapeutic rationale evolve, and any drug development strategy must account for where within that spectrum a patient sits. In early compensated cirrhosis, before clinically significant portal hypertension develops (hepatic venous pressure gradient below 10 mmHg), the dominant driver remains ongoing hepatocellular injury and active fibrogenesis, and the liver retains capacity for fibrosis reversal [52]. As cirrhosis advances and portal hypertension worsens, the pathophysiology shifts: sinusoidal capillarisation, intrahepatic vascular remodelling, splanchnic vasodilation, and hyperdynamic circulation progressively uncouple clinical deterioration from ongoing metabolic hepatocellular injury. In this later stage, agents acting primarily on metabolic drivers or extracellular matrix remodelling may have attenuated clinical benefit within realistic trial timeframes, while mechanisms that address the vascular and hemodynamic components of portal hypertension may be more impactful. Safety considerations also shift, as patients with more advanced cirrhosis have narrower margins for tolerating adverse effects such as gastrointestinal symptoms, weight loss, or changes in hepatic hemodynamics.

The PREDESCI trial illustrates this principle: in patients with compensated cirrhosis and clinically significant portal hypertension, non‐selective beta‐blockers reduced first decompensation by approximately half, driven largely by a reduction in ascites, a benefit achieved through modulation of portal pressure rather than any effect on the underlying fibrotic or metabolic disease [53]. This demonstrates that, in advanced compensated cirrhosis, clinically meaningful outcomes can be achieved by targeting hemodynamic consequences of disease independently of fibrosis regression. The appropriate pharmacological strategy for cirrhosis may therefore need to be tailored to the stage of disease and the magnitude of portal hypertension, rather than fibrosis burden alone. Combinations approaches pairing metabolic and antifibrotic agents with hemodynamically active therapies may ultimately prove necessary.

Full approval in compensated cirrhosis will ultimately require demonstration of benefit on outcomes that matter to patients, including prevention of decompensation, liver transplantation, hepatocellular carcinoma, and death. However, no accelerated approval pathway currently exists for cirrhosis, and this has hindered the ability to evaluate potentially disease‐modifying therapies within feasible trial timelines. Given the relatively low absolute event rates and long latency to clinical events in well‐compensated F4 disease, exclusive reliance on MALO as the primary endpoint may render confirmatory trials prohibitively large and long. Future consideration of reasonably likely surrogate endpoints in cirrhosis, including histological or non‐invasive measures of disease modification that are linked to clinical outcomes, could provide a more feasible path forward. Composite or dual‐endpoint strategies that combine fibrosis‐based assessment with clinical events such as first decompensation warrant active consideration by sponsors and regulators, although their role in regulatory approval remains to be clarified. For non‐cirrhotic MASH, prior to ultimately reducing MALO, accelerated approval can be granted on the basis of histological surrogates (MASH resolution or 1 stage reduction in fibrosis) as these are reasonably likely to predict a reduction in MALO. No such pathway currently exists for cirrhosis population. Trials to date have illustrated both the heterogeneity of the cirrhosis population and the paucity of clinical events within realistic trial durations. Those without clinically significant portal hypertension may be more likely to response to treatment, yet least likely to experience a qualifying event, a paradox that would require either very large sample sizes or very prolonged follow up to resolve.

The biology also sets limits that pharmacology cannot simply override. Cross‐linked collagen, sinusoidal capillarisation, and the progressive attrition of hepatocyte regenerative capacity all constrain what is achievable once cirrhosis is established [54]. Analysis of fibrosis improvement rates across Phase 2b and Phase 3 trials consistently shows that responses in F4 patients cluster at a lower ceiling than those seen in F2 to F3 disease, regardless of which mechanism is being targeted [8]. For many patients with well‐established cirrhosis, stabilizing the disease and preventing the first decompensation event may be a more realistic and meaningful therapeutic goal than histological regression.

Against that backdrop, the results of the SYMMETRY trial have been encouraging. Efruxifermin did not meet its primary endpoint of fibrosis improvement at 36 weeks, but at 96 weeks, 29% of patients in the 50 mg group achieved fibrosis improvement without worsening of MASH in the ITT analysis versus 11% receiving placebo, with consistent improvements in non‐invasive markers of fibrosis, portal hypertension risk, and digital pathology quantification of collagen burden [55]. It is the first evidence that the fibrotic architecture of the compensated cirrhotic liver can be reversed pharmacologically. Consistent with this, a recent network meta‐analysis of pharmacological therapies in compensated MASH cirrhosis identified efruxifermin as the only intervention to date with a statistically significant fibrosis‐improvement signal versus placebo in this population [56].

The semaglutide experience offers a contrasting and sobering lesson. In a randomised Phase 2 trial of 71 patients with MASH‐related compensated cirrhosis, semaglutide 2.4 mg once weekly did not improve fibrosis: only 10.6% of treated patients achieved the primary endpoint versus 29.2% receiving placebo [57]. Metabolic parameters and MASH resolution rates did improve, which is consistent with what the drug does systemically. While the failure on fibrosis is informative, the study was likely underpowered and of insufficient duration to confidently illustrate its potential impact on fibrosis. In a liver that has already become cirrhotic, an approach that works primarily through weight loss and systemic metabolic correction may simply not be able to drive meaningful extracellular matrix remodelling within a 48‐week trial window.

The situation with resmetirom is more nuanced. The FDA prescribing information does report a 19‐fold increase in drug exposure in patients with severe hepatic impairment, which underpins the contraindication in decompensated cirrhosis. But in patients with mild hepatic impairment, which encompasses well‐compensated Child‐Pugh A cirrhosis, the increase in AUC is only 1.3‐fold over that seen in non‐cirrhotic patients. That distinction matters, because the MAESTRO‐NASH‐OUTCOMES trial is specifically enrolling Child‐Pugh A patients, and its results, expected in 2027, will be the first properly powered dataset to show whether resmetirom's histological benefits in F2 to F3 disease translate into event‐level outcomes in the cirrhotic population.

Until those data arrive, management of compensated MASH cirrhosis follows guideline‐directed principles: optimisation of metabolic risk factors, hepatocellular carcinoma surveillance, variceal screening and prophylaxis where indicated, and enrolment in clinical trials wherever possible.

## 6 Beyond the Biopsy: Non‐Invasive Assessment of Fibrosis and Treatment Response

Liver biopsy has occupied a central and contentious role in MASH drug development since the field's inception. However, the non‐invasive test landscape has matured substantially over the past decade. Vibration‐controlled transient elastography measures liver stiffness as a physical property of hepatic tissue and has accumulated independent prognostic data linking both baseline values and longitudinal changes to MALO in large real‐world cohorts [14, 58]. The enhanced liver fibrosis score, a composite of circulating markers of extracellular matrix turnover including hyaluronic acid, procollagen III amino terminal peptide, and tissue inhibitor of metalloproteinase 1, similarly predicts clinical outcomes, particularly in the context of advanced fibrosis where it is approved as a prognostic biomarker to predict progression to fibrosis and hepatic decompensation. MRI‐derived proton density fat fraction has become the standard quantitative steatosis measure in clinical trials, valued for its precision and reproducibility, with threshold changes predictive of histological improvement (particularly MASH resolution) [59, 60, 61]. Magnetic resonance elastography offers a continuous and highly reproducible assessment of liver stiffness that outperforms ultrasound‐based methods, particularly in patients with obesity.

The most significant development in this space, however, is not any individual biomarker but a specific regulatory signal. In August 2025, the FDA accepted the first letter of intent from Echosens to begin formally qualifying VCTE‐derived liver stiffness measurement (Fibroscan) as a reasonably likely surrogate endpoint for accelerated approval in non‐cirrhotic MASH with F2 to F3 fibrosis. In December 2025, a second acceptance followed for magnetic resonance elastography (Resoundant). Much remains to be done, but such letters of intent are the first step of the FDA's Drug Development Tool qualification process, which requires demonstration of biological plausibility, analytical validity, and epidemiological evidence that biomarker changes predict outcomes that matter to patients. While the qualification process will take time, this represents a decisive shift in drug development that will ultimately reduce the need for liver biopsy as a surrogate for efficacy.

The most concrete expression of that response is the joint tirzepatide and retatrutide Phase 3 MASH program registered in September 2025, which eschews liver biopsy almost entirely. The trial uses ELF score and VCTE as both the inclusion criteria for at‐risk MASH and as secondary endpoints at 104 weeks, with the primary outcome defined by clinical events at approximately 224 weeks. By removing requirement for biopsy from the eligibility process, the design dramatically reduces screen failure rates, accelerates enrollment, and allows recruitment of a far larger and more representative patient population than any biopsy‐gated trial has been able to achieve. It also establishes a template that other sponsors are likely to follow as the NIT qualification pathway progresses.

Digital pathology using artificial intelligence occupies a distinct and complementary role in this landscape, one that is sometimes conflated with non‐invasive testing but is mechanistically quite different. AI‐based quantitative assessment of liver histology, using platforms such as qFibrosis and second harmonic generation microscopy, still requires a biopsy sample. Its value lies not in replacing biopsy in clinical practice but in extracting far more information from the biopsy that is taken. In early‐phase development, where decisions about dose selection, mechanism of action, and combination therapy design are being made, AI‐based continuous quantification of collagen burden offers sensitivity to subtle pharmacological signals that categorical NAS and Metavir staging systems cannot resolve [26].

## 7 Therapeutic Horizon: Combination Strategies, the Pipeline, and the Road to Full Approval

### 7.1 The Limits of Monotherapy and the Rationale for Combination

The histological response rates achieved by approved and late‐phase investigational agents, while statistically significant and likely clinically meaningful, are unlikely to be sufficient in isolation to bend the arc of disease burden. Fibrosis improvement in the range of 12% to 15% over placebo at one to two years, however reproducible across trials, means that the majority of treated patients do not achieve histological benefit within current trial durations. Network meta‐analyses of fold change in fibrosis improvement versus placebo across the Phase 2b and 3 landscape suggest the existence of a biological ceiling for single‐agent approaches, likely reflecting the redundancy of fibrogenic and inflammatory pathways in MASH and the impossibility of any single mechanism addressing the full spectrum of metabolic, hepatocellular, and stromal drivers operating simultaneously in advanced disease [62].

### 7.2 Rational Combination Strategies

The most mechanistically coherent and clinically immediate combination pairs resmetirom, which restores intrahepatic lipid homeostasis through direct THRβ‐mediated hepatocellular activity, with a GLP1‐based therapy, which reduces hepatic substrate delivery through systemic metabolic correction. The two mechanisms operate at distinct biological nodes, show no pharmacokinetic interaction of concern, and address complementary aspects of disease pathogenesis. Preclinical studies have demonstrated additive reductions in hepatic fat and preservation of lean mass when THRβ agonists are combined with incretin‐based agents.

FGF21 analogues are particularly well positioned as combination partners given their dual activity across both systemic metabolic and intrahepatic antifibrotic pathways. Three FGF21 analogues are now in advanced development, each acquired by a major pharmaceutical company in 2025 in transactions that signal substantial field‐wide conviction. The concept of an induction strategy using a potent FGF21 analogue to achieve rapid metabolic and antifibrotic reprogramming, followed by maintenance with an approved agent, merits prospective evaluation.

### 7.3 What Full Approval Will Require

Both resmetirom and semaglutide hold accelerated or conditional approval on the basis of histological surrogate endpoints. Confirmation of clinical benefit, and the transition to full approval, requires demonstration of meaningful impact on hard outcomes: prevention of progression to cirrhosis, reduction in hepatic decompensation events, liver transplantation, and all‐cause mortality. These events accrue slowly in a non‐cirrhotic population with compensated disease, which is precisely why the accelerated pathway was created and why the confirmatory trials are structured as long‐term event‐driven studies rather than fixed follow‐up programs.

Both resmetirom and semaglutide trials will provide the longitudinal non‐invasive test data needed to validate biomarker‐outcome relationships, which in turn support the NIT qualification pathway that could eventually replace biopsy in future pivotal trials.

The qualification of non‐invasive tests as reasonably likely surrogate endpoints for accelerated approval, currently in the early stages of the FDA's Drug Development Tool process following acceptance of letters of intent for VCTE and MRE, would fundamentally alter the economics and feasibility of MASH drug development if successful. Lower screen failure rates, shorter recruitment timelines, more representative patient populations, and the ability to monitor treatment response longitudinally without serial biopsy would collectively reduce the cost and duration of Phase 3 development and expand the number of agents that can realistically be brought to registration.

## 8 Conclusion

The approvals of resmetirom and semaglutide establish that hepatic fibrosis in MASH is pharmacologically reversible. Whether that reversal will translate into reduced decompensation, transplantation, and death at a population level remains to be demonstrated. In a disease where fewer than 6% of patients have been diagnosed and fewer than 1% are being treated, the gap between proof of concept and epidemiological impact is substantial and largely independent of drug efficacy. The arc of liver fibrosis in MASH has not yet bent but the tools to bend it are, for the first time, within reach.

## Author Contributions

**Malek Ayoub:** writing – original draft, conceptualization. **Tsubasa Tsutsumi:** writing – original draft, conceptualization. **Mary E. Rinella:** conceptualization, writing – review and editing, supervision, validation.

## Funding

The authors have nothing to report.

## Data Availability

Data sharing not applicable to this article as no datasets were generated or analysed during the current study.
